# Plant-based oral care product exhibits antibacterial effects on different stages of oral multispecies biofilm development in vitro

**DOI:** 10.1186/s12903-021-01504-4

**Published:** 2021-04-01

**Authors:** Nadine Kommerein, Almut Johanna Weigel, Meike Stiesch, Katharina Doll

**Affiliations:** 1 Department of Prosthetic Dentistry and Biomedical Materials Science, Hannover Medical School, Carl-Neuberg-Str. 1, 30625 Hannover, Germany; 2 Department of Prosthetic Dentistry and Biomedical Materials Science, Lower Saxony Center for Biomedical Engineering, Implant Research and Development (NIFE), Hannover Medical School, Stadtfelddamm 34, 30625 Hannover, Germany

**Keywords:** Periodontal pathogenic bacteria, Oral biofilm, Multispecies biofilm, Oral hygiene, Naturopathic oral care product, Medicinal plant extracts, Essential oils, Minimum inhibitory concentration (MIC), Minimum biofilm inhibitory concentration (MBIC), Minimum biofilm eradication concentration (MBEC)

## Abstract

**Background:**

Excessive biofilm formation on surfaces in the oral cavity is amongst the main reasons for severe infection development like periodontitis and peri-implantitis. Mechanical biofilm removal as well as the use of adjuvant antiseptics supports the prevention of pathogenic biofilm formation. Recently, the antibacterial effect of the oral care product REPHA-OS^®^, based on medicinal plant extracts and essential oils, has been demonstrated on oral pathogens grown on agar plates. In the present study, the effectiveness of the product on medical relevant oral biofilm development should be demonstrated for the first time.

**Methods:**

An established in vitro oral multispecies biofilm, composed of *Streptococcus oralis, Actinomyces naeslundii, Veillonella dispar* and *Porphyromonas gingivalis,* was used to analyze the antibacterial effect of different REPHA-OS^®^ concentrations on planktonic bacteria, biofilm formation and mature biofilms. It was quantified using metabolic activity assays and live/dead fluorescence staining combined with three-dimensional confocal laser-scanning microscopy. Additionally, effects on species distribution inside the biofilm were assessed by means of quantitative real-time PCR.

**Results:**

REPHA-OS^®^ showed statistically significant antimicrobial effects on all stages of biofilm development: a minimal inhibitory concentration of 5% could be detected for both, for planktonic bacteria and for biofilm formation. Interestingly, only a slightly higher concentration of 10% was necessary to completely kill all bacteria in mature biofilms also. In contrast, an influence on the biofilm matrix or the species distribution could not be observed. The effect could be attributed to the herbal ingredients, not to the contained ethanol.

**Conclusion:**

The strong antibacterial effect of REPHA-OS^®^ on different stages of oral biofilm development strengthens its application as an alternative adjuvant in oral care therapies.

**Supplementary Information:**

The online version contains supplementary material available at 10.1186/s12903-021-01504-4.

## Background

The human oral microbiome comprises more than 700 different species of bacteria [1] and thus is one of the most diverse bacterial habitats in the human body. Bacteria colonize hard and soft tissue by forming highly complex structured biofilms, also known as dental plaque [2]. These biofilms are defined as attached bacterial multispecies agglomerates that are surrounded by a self-produced matrix made from extracellular polymeric substances, e.g., secreted DNA, polysaccharides and/or proteins and exhibit a biofilm-specific gene expression pattern [2]. The development of oral biofilms is a multistep process. First, pioneer bacteria attach to the proteinaceous pellicle covering all oral surfaces by species-specific adhesion mechanisms [3, 4]. Dominant species in this context are streptococci, like *Streptococcus oralis* [5, 6]. Other initial colonizers that co-aggregate with *S. oralis* are *Actinomyces naeslundii* and *Veillonella dispar* [5, 6]. These bacteria belong to the commensal oral microbiome and are associated with periodontal health. Oral pathogens, like *Porphyromonas gingivalis*, can be found only in minor contribution in the initial biofilm [6]. However, if the bacterial biomass excessively increases, e.g., due to reduced oral hygiene or systemic precondition, the bacterial species distribution shifts and the amount of pathogens increases [7]. These dysbiotic biofilms interfere with the host immune system and are often the reason for severe local infections like periodontitis and peri-implantitis [8]. Treatment of these oral infections is difficult due to the specific properties of biofilms: the biofilm matrix serves as diffusion barrier for antibacterial substances and the altered gene expression pattern effectively protects against the immune response of the host [9]. Therefore, prevention of an uncontrolled growth of bacteria is essential for prevention of infections in the oral cavity.

Mechanical biofilm removal by daily tooth brushing as well as the use of antiseptic mouth rinsing solutions support the maintenance of a healthy oral microflora to inhibit the formation of pathogenic biofilms [10]. Especially for patients with reduced manual abilities, e.g., due to advanced age, the use of adjuvant antiseptics on a daily basis can help to optimize oral hygiene [11]. Ideally, oral antiseptics should reduce bacterial colonization without impairing oral homeostasis or inducing bacterial resistance [12]. However, increasing bacterial resistance to common oral antiseptics could be reported [13] and studies also indicate a possible cross-resistance between antibiotics and chemical antiseptics, like chlorhexidine [14, 15]. This enhances the need for alternative oral care products.

The plant-based oral mouth spray REPHA-OS^®^ (Repha GmbH Biologische Arzneimittel, Langenhagen, Germany) for preventive oral care contains extracts of *Potentilla erecta* root (bloodroot), *Krameria triandra* root (rhatany), *Commiphora myrrha* resin (myrrh) as well as essential oils of *Eugenia caryophyllus* flower (clove), *Mentha piperita* (peppermint), *Eucalyptus globulus* leaf (eucalyptus), *Pimpinella anisum* fruit (anise) and the additives limonene, linalool, levomenthol, and stevia as well as 69% ethanol as solvent. Antibacterial effects of the individual ingredients are well established [16–20]. Recently, the combination product was shown to effectively reduce colony growth of several different oral pathogens (amongst them *P. gingivalis*) in an agar dilution assay [21]. A minimally inhibitory concentration of 5–10%, depending on the bacterial strain, could be identified.

Based on the aforementioned data, we hypothesize that REPHA-OS^®^ exhibits antibacterial effects on medical relevant biofilms of the oral cavity as well. To address this hypothesis, different stages of an established in vitro oral multispecies biofilm [22], composed of *S. oralis, A. naeslundii, V. dispar* and *P.* *gingivalis*, were treated with the product in different concentrations. By using metabolic activity assays and live/dead fluorescence staining followed by confocal laser-scanning microscopy, the antibacterial effect could be confirmed and the minimal inhibitory concentration (MIC) on planktonic cultures, the minimal biofilm inhibitory concentration (MBIC) on biofilm formation, and the minimal biofilm eradication concentration (MBEC) on mature biofilms (24 h old) identified. Additionally, the effect of REPHA-OS^®^ on the species distribution in the model biofilm was shown by quantitative real-time PCR.

## Methods

### Bacterial strains and growth conditions

For evaluation of antibacterial effects, the established oral multispecies biofilm by Kommerein et al. [22] was used. Bacterial cultures were obtained from the German Strain Collection for Microorganisms and Cell Cultures (DSMZ; Braunschweig, Germany) and the American Type Culture Collection (ATCC; Manassas, USA). All strains are listed in Table 1. Bacteria were pre-cultured in Brain Heart Infusion Medium (BHI; Oxoid, Wesel, Germany) containing 10 µg/ml vitamin K (Roth, Karlsruhe, Germany) under anaerobic conditions at 37 °C for 24 h. After pre-cultivation, each species was adjusted to a final optical density at 600 nm (OD_600_) of 0.01 in BHI + vitamin K and then they were combined in equal volumes. This bacterial suspension was mixed with the respective test solutions, directly seeded into multiwell plates and cultivated as described below in detail (see also Table 2).

**Table 1:** Overview of the used bacterial strains

Species	Strain
*Streptococcus oralis *(*S. oralis)*	ATCC^®^ 9811
*Actinomyces naeslundii *(*A. naeslundii)*	DSM 43013
*Veillonella dispar *(*V. dispar)*	DSM 20735
*Porphyromonas gingivalis *(*P. gingivalis)*	DSM 20709

**Table 2:** Overview of the applied REPHA-OS^®^ (and 69% ethanol) concentrations, exposure times and cultivation conditions at 37 °C under anaerobic conditions

Variations	(I) planktonic bacteria	(II) biofilm formation	(III) mature biofilms (24 h old)	(IV) mature biofilms (24 h old)
REPHA-OS^®^ (and ethanol) concentrations	1.25% (0.8625%)	–	–	–
	2.5% (1.725%)	2.5% (1.725%)	–	–
	5% (3.45%)	5% (3.45%)	5% (3.45%)	5% (3.45%)
	10% (6.9%)	10% (6.9%)	10% (6.9%)	–
	–	–	50% (34.5%)	–
Exposure time	24 h	24 h	2 h	5 min
Cultivation conditions	Under rotation (500 rpm)	Static	Static	Static

### Investigation of the antibacterial effect of REPHA-OS^®^ on different stages of multispecies biofilm formation

REPHA-OS^®^ was received as a production bottling of the manufacturer Repha GmbH (Langenhagen, Germany). The product contains *Potentilla erecta* root (bloodroot), *Krameria triandra* root (rhatany), *Commiphora myrrha* resin (myrrh), *Eugenia caryophyllus* flower (clove), *Mentha piperita* (peppermint), *Eucalyptus globulus* leaf (eucalyptus), *Pimpinella anisum* fruit (anise), limonene, linalool, levomenthol, stevia and 69% ethanol. To determine the antibacterial effect of REPHA-OS^®^, it was added in varying concentrations to (I) planktonic bacteria for 24 h, (II) biofilm formation for 24 h, and already 24-h old, mature biofilms (24 h old) for (III) two-hours incubation and (IV) five-minute incubation. Identical test procedures were also carried out with corresponding dilutions of 69% ethanol as ethanol control. As a growth control, the bacteria were cultivated without addition of REPHA-OS^®^ or ethanol (hereinafter referred to as "BHI/VitK"). In order to exclude possible dilution effects, lower concentrations were filled with water to the volume of the highest concentration. To evaluate the effect of diluting the medium, a reduced media control (hereinafter referred to as "BHI/VitK red") diluted with water correspondingly to the volume of the highest concentration was also carried along. Cultivation of bacteria with REPHA-OS^®^ and ethanol was performed at 37 °C under anaerobic conditions for (I) planktonic bacteria under rotation (500 rpm), (II) biofilm formation statically for 24 h, and mature biofilms (24 h old) were incubated with REPHA-OS^®^ and Ethanol for (III) 2 h and (IV) for 5 min under static conditions. The experiments were performed in three biological replicates (= independent precultures) with three (planktonic bacteria) or two (biofilms) technical replicates (= different wells), each. An overview of the used concentrations, exposure times and cultivation parameters is given in Table 2.

### Analysis of planktonic cultures—bacterial growth and metabolic activity

To determination the influence of REPHA-OS^®^ and ethanol on bacterial growth of planktonic cultures, the optical density (absorption) was measured at 600 nm (OD_600_) with a plate reader (Tecan, Mennedorf, Switzerland). To show the effects on metabolic activity, the BacTiter-Glo™ Microbial Viability Assay (Promega, Mannheim, Germany) was used to quantify the amount of adenosine triphosphate (ATP). BacTiter-Glo™ reagent was prepared according to the manufacturer's instructions and then mixed 1:1 with the bacterial suspension. After 5 min incubation at room temperature, luminescence was measured again with a plate reader (Tecan, Mennedorf, Switzerland). All results were normalized to the full medium control (BHI/VitK).

### Analysis of biofilms—metabolic activity, biofilm volume and live/dead distribution

To investigate the influence of REPHA-OS^®^ and ethanol on biofilm formation and mature biofilms (24 h old), metabolic activity was determined by resazurin assay, and biofilm volume and live/death distribution by fluorescence staining and subsequent confocal laser-scanning microscopy (CLSM). First, biofilms were washed twice with phosphate buffered saline (PBS) to remove non-adherent bacteria. Afterwards, they were incubated with 0.001% resazurin (Sigma Aldrich, Darmstadt, Germany) in PBS for 45 min statically under anaerobic conditions at 37 °C. To measure the metabolic activity, 100 µl each were pipetted into a black 96 well plate. Fluorescence was measured using a plate reader (Tecan, Mennedorf, Switzerland) with an excitation wavelength of 530 nm and an emission wavelength of 590 nm. All results were normalized to the full medium control (BHI/VitK). Fluorescent staining of biofilms was done after metabolic activity was determined by resazurin assay. The resazurin solution was removed and biofilms were stained by adding SYTO^®^9 and propidium iodide (LIVE/DEAD^®^ BacLight™ Bacterial Viability Kit, Life Technologies, Carlsbad, California, USA) for 15 min according to the manufacturer's instructions. Subsequently, samples were fixed in PBS with 2.5% glutardialdehyde solution (Roth, Karlsruhe, Germany) for 15 min. Biofilms were covered with PBS and analyzed with a confocal laser scanning microscope (CLSM; Leica TCS SP8, Leica Microsystems, Mannheim, Germany). SYTO^®^9 dye was excited at 488 nm and emission was detected at a wavelength of 500–550 nm; propidium iodide was excited at 552 nm and emission was measured at 675–750 nm. Five images were taken from each biofilm with a z-step size of 5 µm. Evaluation of biofilm volume and live/dead distribution was performed with Imaris × 64 8.4 software (Bitplane AG, Zurich, Switzerland).

### Analysis of species distribution by PMA treatment, DNA isolation and qRT-PCR

For control biofilms (BHI/VitK) and mature biofilms (24 h old) treated with REPHA-OS^®^ for 5 min and 2 h, the species distribution of the multispecies biofilm was analyzed. Biofilms were collected after cultivation/treatment by scraping and treated with propidium monoazide (PMA) to selectively examine viable bacteria only as described by Kommerein et al. [22], except that PMA was added at a final concentration of 120 μM. Bacterial DNA was isolated using the FastDNA™ SPIN Kit for Soil (MP Biomedicals, Eschwege, Germany) and quantified by spectrophotometric measurement (Nanodrop 2000, Thermo Fisher Scientific Inc., Waltham, Massachusetts, USA). To obtain sufficient amount of DNA, for control biofilms samples of each biological replicate, and for treated biofilms all samples, were pooled. Quantitative real-time PCR (qRT-PCR) was performed using the iQ5 real time PCR detection system (Bio-Rad, Hercules, California, USA) for each pooled sample in duplicates. Reaction components (Supplementary Table S1), primer pairs (Supplementary Table S2) and cycle conditions (Supplementary Table S3) were used according to previous works [22, 23] and are listed in the supporting information. For each bacterial species, a standard curve was carried along that allowed for the calculation of genomic DNA for each species in the biofilm samples. By dividing the amount of DNA by the theoretical genome weight per cell (Supplementary Table S4), the number of bacterial cells per ng DNA could be calculated.

### Statistical analysis

Statistical analysis and graphic processing of data was carried out with GraphPad Prism Software 8.4 (GraphPad Software Inc., La Jolla, USA). For experiments on planktonic bacteria, data were checked for normal distribution using D'Agostino-Pearson Omnibus normality test. Subsequently, significant differences to the control (BHI/Vit K red), or between REPHA-OS^®^ and the respective ethanol concentration, were determined by Kruskal–Wallis test with Dunn's correction for multiple comparisons. All biofilm experiments were checked for normal distribution by Kolmogorov–Smirnov normality test. Subsequently, significant differences to the control (BHI/Vit K red), or between REPHA-OS^®^ and the respective ethanol concentration, were determined using Ordinary One-Way ANOVA with Bonferroni's correction for multiple comparisons. To compare differences between REPHA-OS^®^ and the respective ethanol concentration after short incubation time of 5 min, Mann–Whitney test was used. The significance level was set to p ≤ 0.05 for all comparisons.

## Results

### Antibacterial effect of REPHA-OS^®^ on planktonic bacteria

**Fig. 1:**
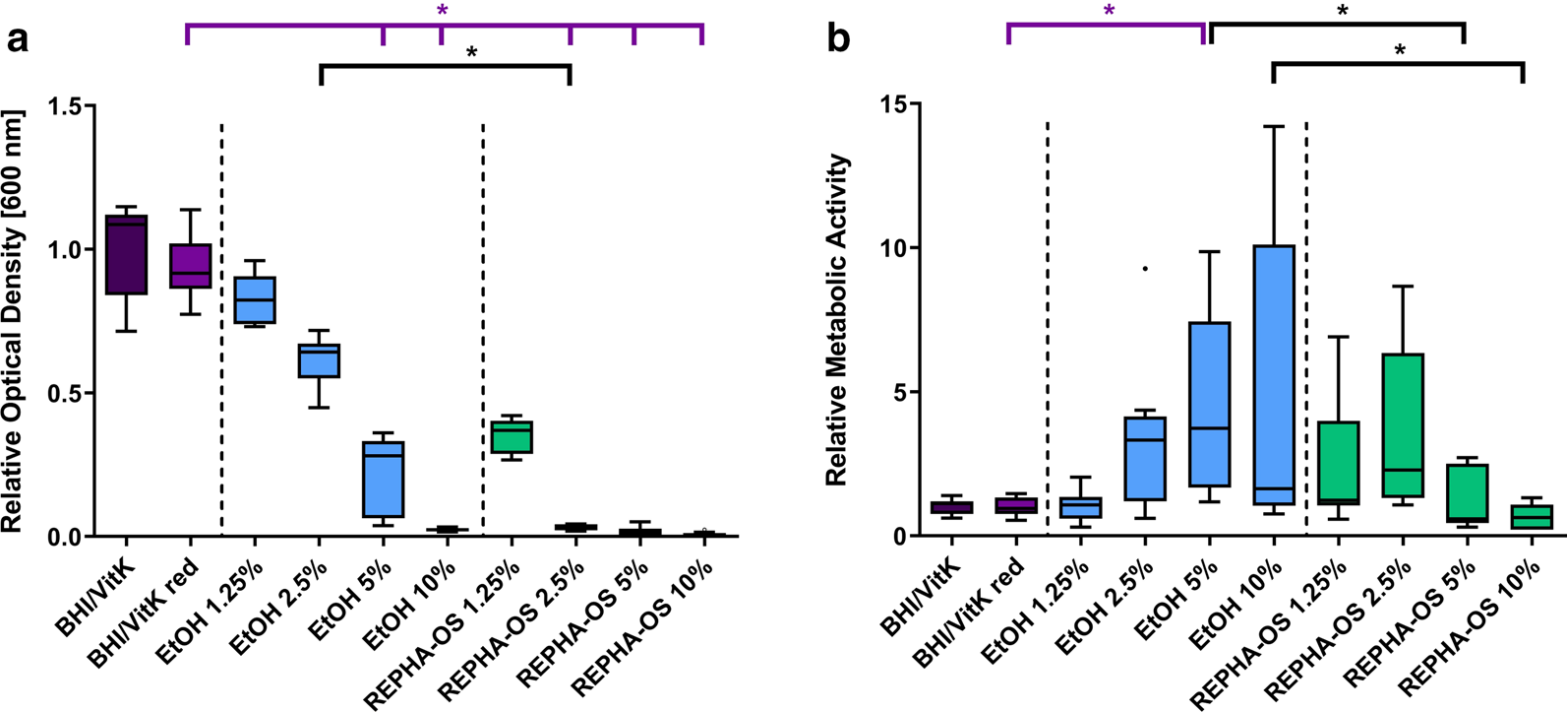
Antibacterial effect of REPHA-OS^®^ on planktonic bacteria. **a** Tukey Box Plots of optical density and **b** metabolic activity determined by BacTiter-Glo™ Assay of planktonic bacteria of the oral multispecies biofilm after 24 h incubation with REPHA-OS^®^ or the corresponding ethanol concentration. Purple brackets show significant differences to the BHI/VitK red control and black brackets show significant differences between REPHA-OS^®^ and the respective ethanol concentration, each at a significance level of p ≤ 0.05

The effect of REPHA-OS^®^ on planktonic bacteria after 24-h incubation was investigated by measuring the optical density to determine bacterial growth (Fig. 1a) and BacTiter-Glo™ assay to quantify the amount of ATP as parameter for metabolic activity (Fig. 1b). Dilution of growth medium (BHI/VitK red), compared to full medium (BHI/VitK), had no significant effect, neither on growth nor on metabolic activity of planktonic bacteria.

Both Ethanol and REPHA-OS^®^ reduced bacterial growth compared to the control (BHI/VitK red), but from different concentrations (Fig. 1a). Compared to the corresponding ethanol controls, bacteria showed significantly less growth under the influence of 2.5% REPHA-OS^®^ (p = 0.0384). The metabolic activity of bacteria in both medium controls and under influence of ethanol at a concentration corresponding to 1.25% REPHA-OS^®^ was comparably low. While an increasing metabolic activity could be observed with raising ethanol concentrations, with increasing REPHA-OS^®^ concentrations metabolic activity decreased to a level comparable to the medium controls (Fig. 1b). At concentrations of 5% and 10% REPHA-OS^®^, metabolic activity was significantly lower than in the corresponding ethanol control (p = 0.0027 and p = 0.0050, respectively).

The lowest dilution level, where no bacterial growth was detectable and the metabolic activity was significantly reduced compared to the ethanol control, was declared as the minimum inhibitory concentration (MIC). Based on the results, a MIC of 5% REPHA-OS^®^ could be identified for planktonic bacteria (Table 3).

**Table 3:** Determined inhibitory concentrations of REPHA-OS^®^ and 69% ethanol

Inhibitory concentration	Abbreviation	REPHA-OS^®^	69% ethanol
Minimum inhibitory concentration	MIC	5%	> 10%
Minimum biofilm inhibitory concentration	MBIC	5%	> 10%
Minimum biofilm eradication concentration	MBEC	10%	> 50%

### Antibacterial effect of REPHA-OS^®^ on biofilm formation

**Fig. 2:**
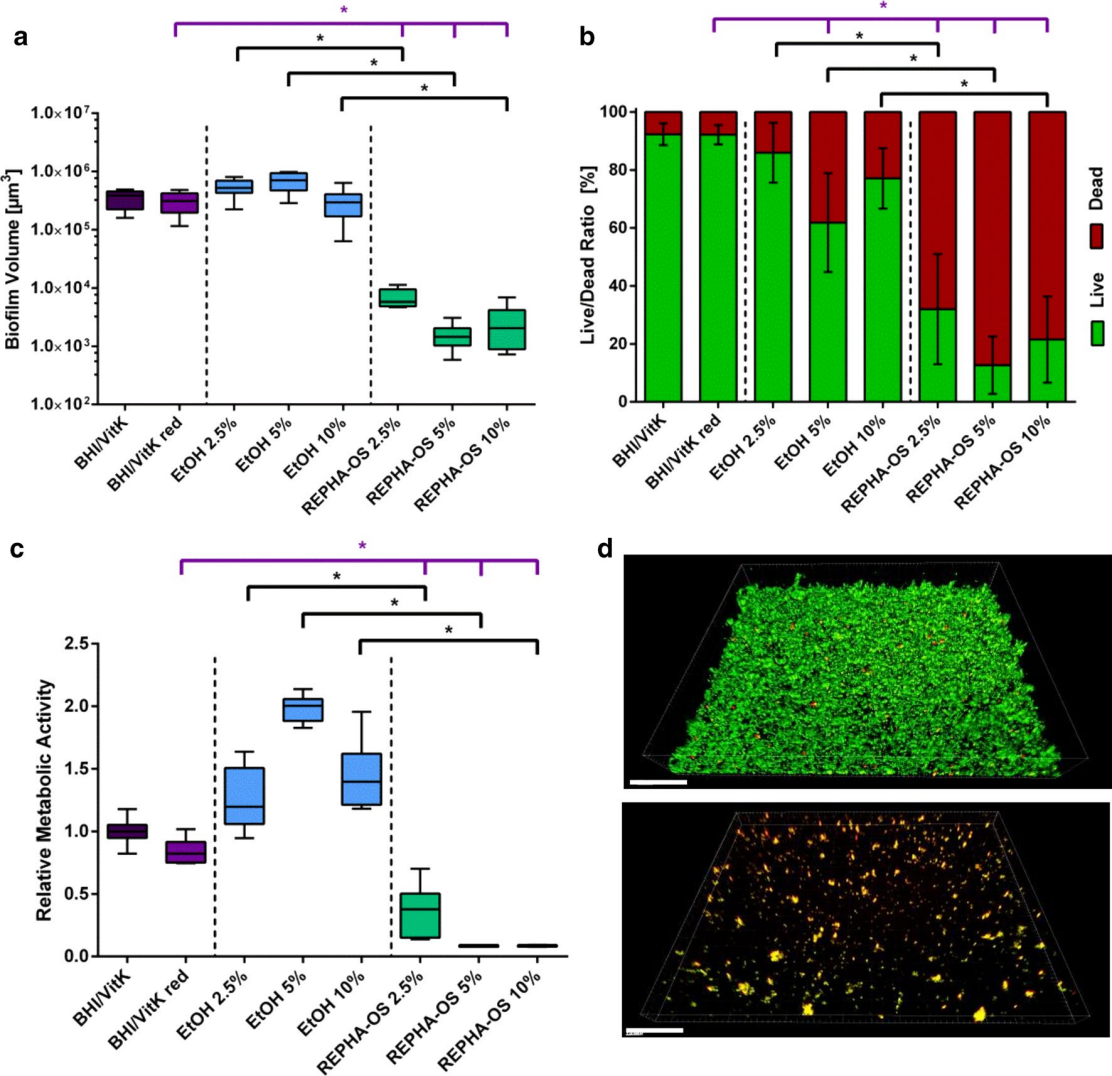
Antibacterial effect of REPHA-OS^®^ on biofilm formation. **a** Tukey Box Plots of biofilm volume, **b** mean value ± standard deviation of the live/dead distribution and **c** Tukey Box Plots of metabolic activity determined by resazurin assay of bacteria in biofilm after 24 h incubation with REPHA-OS^®^ or the corresponding ethanol concentration. Purple brackets show significant differences to the BHI/VitK red control and black brackets show significant differences between REPHA-OS^®^ and the corresponding ethanol concentration, each at a significance level of p ≤ 0.05. **d**—top Control biofilm (BHI/VitK) and **d**—bottom biofilm incubated with 5% REPHA-OS^®^ after 24-h growth, live/dead fluorescence staining and subsequent confocal laser scanning microscopy. Viable bacteria are shown in green, dead bacteria in red/yellow. The 3D reconstruction was created with the software Bitplane Imaris. Scale bar = 50 µm

Based on the MIC of 5% REPHA-OS^®^ for planktonic bacteria, the effect on the formation of an oral multispecies biofilm was analyzed framing at 2.5%, 5% and 10%. The effect of REPHA-OS^®^ on biofilm formation was investigated by analyzing biofilm volume (Fig. 2a), live/dead distribution (Fig. 2b) and by measuring metabolic activity using resazurin assay (Fig. 2c). In this experimental set up as well, dilution of bacterial medium (BHI/VitK red) had no significant effect on biofilm growth, live/dead distribution or normalized activity of bacteria in biofilm compared to full medium (BHI/VitK).

Regarding biofilm volume, no effect of ethanol treatment at any concentration could be observed, whereas biofilm volume of REPHA-OS^®^ treated samples was remarkably reduced (Fig. 2a). This was statistically significant compared to the BHI/VitK red control and the corresponding ethanol concentrations (all p ≤ 0.0078). As microscopical images show, for REPHA-OS^®^ treated samples, only separated bacterial clusters rather than a three-dimensional biofilm, could be detected (Fig. 2d). The analysis of live/dead distribution within biofilms also showed a significant increase in the proportion of dead bacteria for all REPHA-OS^®^ concentrations compared to the growth (BHI/VitK red) and the ethanol controls, respectively (Fig. 2b, all p < 0.0001). Quantifying metabolic activity revealed an increase under the influence of ethanol compared to the medium control (BHI/VitK red). When treated with REPHA-OS^®^, in contrast, metabolic activity was significantly lower than those of the medium control (BHI/VitK red), as well as compared to the corresponding ethanol controls (Fig. 2c, all p ≤ 0.0001).

As minimum biofilm inhibitory concentration (MBIC; defined as the lowest concentration required to inhibit biofilm formation), the lowest dilution level was evaluated, at which biofilm volume was significantly reduced, the number of dead cells significantly increased and no metabolic activity was detectable. A MBIC of 5% REPHA-OS^®^ could be determined for forming biofilms (Table 3).

### Antibacterial effect of REPHA-OS^®^ on mature biofilms after two-hour incubation

**Fig. 3:**
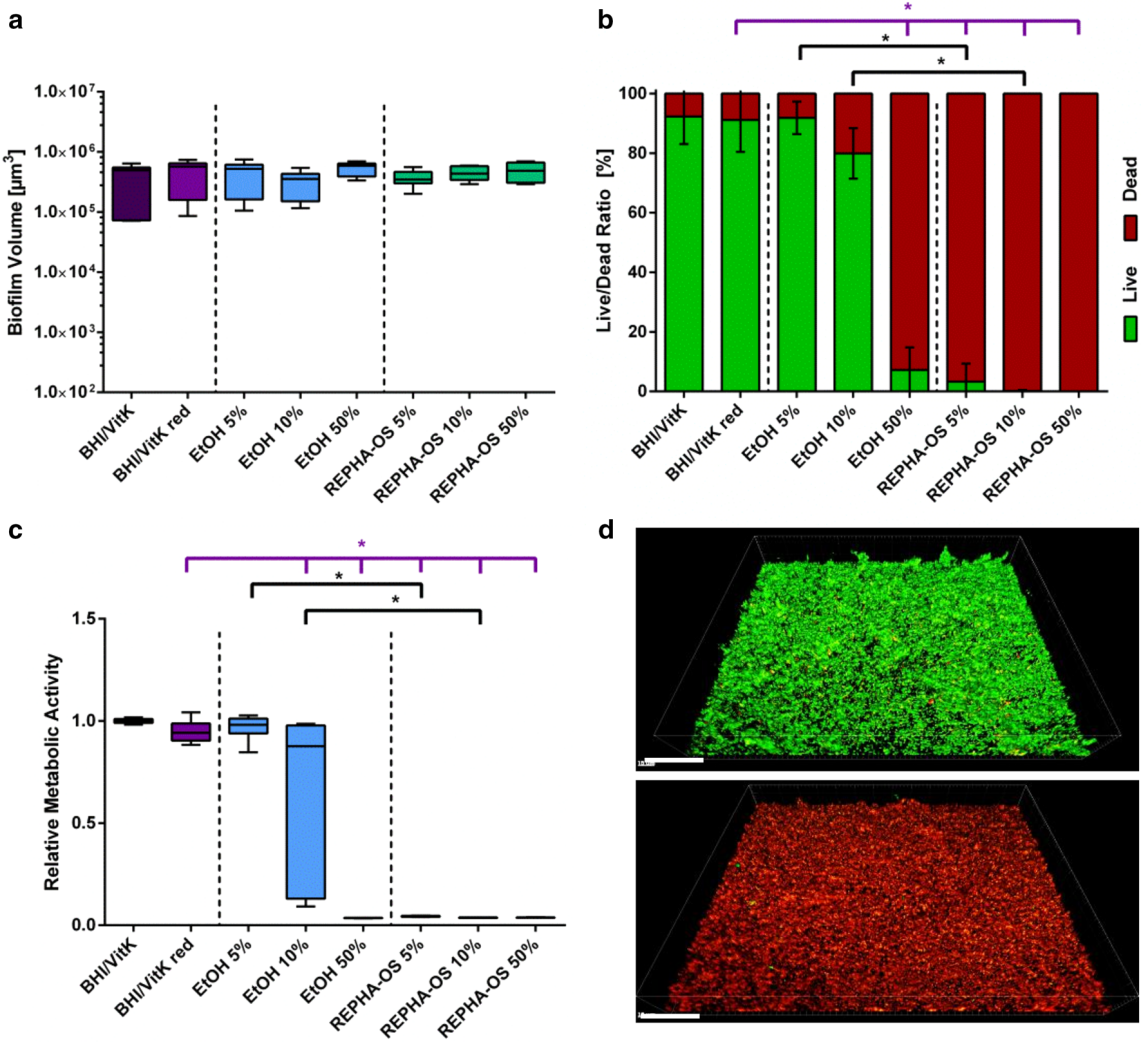
Antibacterial effect of two-hour incubation of REPHA-OS^®^ on mature biofilms. **a** Tukey Box Plots of biofilm volume, **b** mean value ± standard deviation of the live/dead distribution, and **C** Tukey Box Plots of metabolic activity determined by resazurin assay of bacteria in biofilm after two-hour incubation with REPHA-OS^®^ or the corresponding ethanol concentration. Purple brackets show significant decreases to the control (BHI/VitK red) and black brackets show significant differences between REPHA-OS^®^ and the respective ethanol concentration, each at a significance level of p ≤ 0.05. **c**—top Live/dead fluorescence staining of control biofilm after 26-h growth (BHI/VitK) and **d**—bottom biofilm after 26-h growth and REPHA-OS^®^ treatment (24-h growth without and 2 h with 5% REPHA-OS^®^). Viable bacteria are shown in green, dead bacteria in red/yellow. The 3D reconstruction was created with the software Bitplane Imaris. Scale bare = 50 µm

Starting from the previously determined MBIC of 5%, additional 10% and 50% were selected to investigate the antimicrobial effect of two-hour REPHA-OS^®^ treatment on mature biofilms (24 h old). The effect of REPHA-OS^®^ on mature biofilms was also investigated by analyzing biofilm volume (Fig. 3a), live/dead distribution (Fig. 3b), and by measuring metabolic activity using resazurin assay (Fig. 3c). Again, dilution of bacterial medium (BHI/VitK red), compared to full medium (BHI/VitK), had no significant influence on biofilm volume, live/dead distribution, and normalized activity of bacteria in biofilm.

After a two-hour exposure time, REPHA-OS^®^ and also the corresponding ethanol concentration showed no effect on biofilm volume compared to the control (BHI/VitK red; Fig. 3a). In contrast, the percentage of dead cells increased with increasing concentrations and reached statistical significance for ethanol as present in 50% REPHA-OS^®^ and for ≥ 5% REPHA-OS^®^ compared to the control (BHI/ VitK red) and the respective ethanol concentrations (Fig. 3b, all p < 0.0001). Microscopical images confirmed these findings, as live/dead stained biofilms completely turned red (dead) after REPHA-OS^®^ treatment compared to the vital green control biofilms (Fig. 3d). When quantifying metabolic activity of mature biofilms, an increasing dose dependent reduction could be detected that was statistically significant at ethanol concentrations as present in ≥ 10% REPHA-OS^®^ (p ≤ 0.0147) and ≥ 5% REPHA-OS^®^ (all p < 0.0001, Fig. 3c).

The minimum biofilm eradication concentration (MBEC; defined as the lowest concentration required to kill all microorganisms in a biofilm) was defined as the lowest dilution level, at which all bacteria in the biofilm were completely killed and no metabolic activity could be detected. Therefore, a MBEC of 10% REPHA-OS^®^ could be determined (see Table 3).

### Effects of REPHA-OS^®^ on species distribution within biofilms

**Fig. 4:**
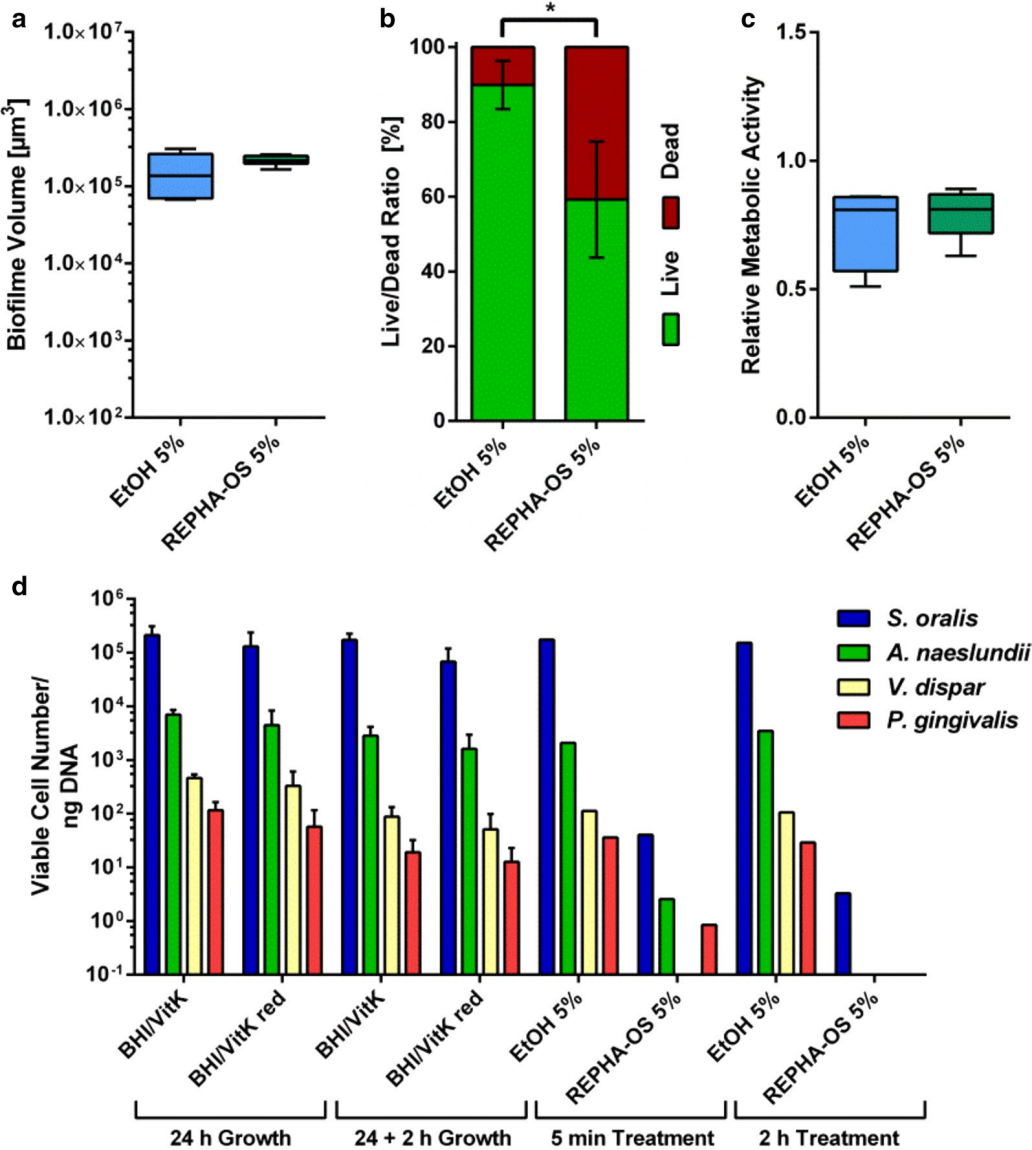
Antibacterial effect of five-minute REPHA-OS^®^ on species distribution in mature biofilms. **a** Tukey Box Plots of biofilm volume, **b** mean value ± standard deviation of the live/dead distribution, and **c** Tukey Box Plots of metabolic activity determined by Resazurin Assay of bacteria in biofilm after five-minute incubation with REPHA-OS^®^ or the corresponding ethanol concentration. Black brackets show significant differences between REPHA-OS^®^ and the respective ethanol concentration, each at a significance level of p ≤ 0.05. **d** Mean value ± standard deviation of viable bacterial species distribution of control biofilms after 24-h and 26-h growth (BHI/VitK and BHI/VitK red) and biofilms after two-hour and five-minute REPHA-OS^®^ treatment and the corresponding ethanol control. As the latter samples had to be pooled, no standard deviation could be calculated

In order to determine the effect of REPHA-OS^®^ on the individual bacterial species distribution, a concentration of 5% (one dilution step below the MBEC) with a reduced incubation time of five minutes was additionally examined on mature biofilms in order to provide an intermediate situation before complete killing of the biofilm. As in the experiment with two-hour incubation periods, first, evaluation of the effect of five minutes REPHA-OS^®^ on mature biofilms was carried out by analyzing biofilm volume (Fig. 4a), live/dead distribution (Fig. 4b) and by measuring metabolic activity by means of a resazurin assay (Fig. 4c). Because of the short incubation time, an additional BHI/VitK or BHI/VitK red control was renounced, and the results were compared to the controls of the previous experiments. Comparing 5% REPHA-OS^®^ and the corresponding ethanol control, no difference regarding biofilm volume and normalized metabolic activity, but a significant difference in live/dead distribution (p = 0.0022) could be detected (Fig. 4a–c).

The individual bacterial species distribution was determined for 24-h old control biofilms grown in both media (BHI/VitK and BHI/VitK red), for 24-h old control biofilms incubated additional two hours in the respective medium, and for 24-h old biofilms treated with 5% REPHA-OS^®^ or the respective ethanol concentration for five minutes or two hours (Fig. 4d). For all control biofilms, equal numbers of viable cells and equal species distributions could be determined. *S. oralis* was the most dominant bacterial species, followed by *A. naeslundii*, *V.* *dispar*, and *P. gingivalis*. For biofilms treated with ethanol in a concentration as present in 5% REPHA-OS^®^ for five minutes or two hours, the same numbers of viable cells and species distribution was detected. When mature biofilms were treated with 5% REPHA-OS^®^ for five minutes, the number of viable cells was clearly reduced. The species distribution was almost maintained with exception of *V. dispar*, which could not be detected anymore. When mature biofilms were further incubated with 5% REPHA-OS^®^ for two hours, viable cells could only be detected for *S. oralis* with additional reduced amounts. For all other species, no viable cells could be detected anymore.

## Discussion

The prevention of biofilm-associated infections of the oral cavity, like periodontal and peri-implant diseases, is usually done mechanically by consistent tooth brushing (toothbrushes, dental floss, interdental brushes) and by adjuvant application of mouth rinsing solutions [24–26]. Due to several known negative side effects of chemical mouth rinsing solutions, e.g., the killing of nitrate-reducing commensal bacteria, which regulate the blood pressure [14, 27–30], there should be a great interest in alternative and/or natural products for plaque control.

In a previous study, we have investigated the effect of REPHA-OS^®^ on colony growth on agar plates of single periodontal pathogens and halitosis-associated bacteria (*Aggregatibacter actinomycetemcomitans*, *Fusobacterium nucleatum*, *Prevotella intermedia, P. gingivalis* and *Solobacterium moorei*) [21]. An antibacterial effect of REPHA-OS^®^ was observed for all investigated oral pathogens, with a maximum MIC of 10%. A similar effect has also been described for several skin- and gut-associated bacterial species [31]. However, none of these studies took into account the physiological biofilm morphology, which is crucial for infection development.

In this study, an antibacterial effect of REPHA-OS^®^ on different developmental stages of oral multispecies biofilms in vitro has been hypothesized. For the application of REPHA-OS^®^, the growth medium (BHI/VitK) had to be diluted. Thus, dilution controls (BHI/VitK red) were included in all experiments. Compared to full medium (BHI/VitK), its dilution (BHI/VitK red) had no significant influence on growth of planktonic bacteria (Fig. 1a) or biofilms (Fig. 2a, 3a) nor on their metabolic activities (Fig. 1c, 2c, 3c) or live/dead distributions within the biofilms (Fig. 2b, 3b). Any antibacterial effect observed could thus not be attributed to a simple reduction in nutrients available. Interestingly, quantification of metabolic activity of the planktonic bacteria revealed a comparatively low metabolic activity of the investigated medium controls (Fig. 1b). Metabolic activity in planktonic cultures was assessed by quantifying ATP. As this molecule is only produced in metabolic active cells and rapidly degraded after cell death [32–35], it can serve as indicator for antibacterial effects. However, bacteria in the early stationary phase are also known to show reduced metabolic activity, and thus a decrease in ATP levels can be observed as well [36, 37]. In correlation to the results of bacterial growth (Fig. 1a), bacteria in planktonic control samples should already have been in the stationary phase. Their low metabolic activity is, thus, not related to an antibacterial effect. This could also be the case for bacteria incubated with ethanol of a concentration as present in 1.25% REPHA-OS^®^.

In order to ensure that an antimicrobial effect of REPHA-OS^®^ is not simply caused by ethanol (REPHA-OS^®^ contains 69% ethanol as solvent), relevant controls were included in all experiments in the appropriate concentrations. With rising concentrations of ethanol, a significant reduction of bacterial growth (Fig. 1a) and an increase in metabolic activity of the planktonic bacteria (Fig. 1b) could be observed. A comparable phenomenon was observed for biofilm formation. In contrast to planktonic cultures, the metabolic activity of biofilms was quantified by direct measurement of respiratory activity using the resazurin assay. The use of this test also for planktonic cultures was not possible due to the reduced pH in these cultures. During biofilm formation, compared to the medium controls (BHI/VitK red), the biofilm volume of the bacteria incubated with ethanol did not change remarkably (Fig. 2a), whereas the metabolic activity increased under the influence of ethanol (Fig. 2c). An increase in metabolic activity can be either attributed to bacterial growth activities, but also to an increased metabolism as part of bacterial stress response [38]. Alcohol *inter alia* alters membrane charges, which induces, e.g., the expression of phage shock and heat shock proteins [39, 40]. In combination with the results of planktonic bacteria growth measurement and biofilm volume quantification, these observations suggest an increase in metabolic activity as part of a stress reaction of the bacteria to ethanol. However, if the ethanol concentration further increases, the metabolic activity gradually decreases—as can be seen for mature biofilms (Fig. 3c)—now exhibiting a toxic reaction. Nevertheless, for planktonic bacteria as well as for biofilm formation and mature biofilms (24 h old), ethanol showed no antimicrobial effect at concentrations as present in the effective REPHA-OS^®^ concentrations (Table 3).

In contrast, REPHA-OS^®^ showed strong antimicrobial effects on all tested developmental stages of oral multispecies biofilms in vitro: planktonic bacteria (MIC of 5%), biofilm formation (MBIC of 5%) and mature biofilms (24 h old; MBEC of 10%). Thus, the hypothesis of this study could be thoroughly confirmed. The product contains medicinal plant extracts and essential oils. For the single ingredients, a toxic effect has already been described [16–20]. Polyphenolic tannins from bloodroot and ratanhia impair bacterial metabolism and nutrient uptake, which leads, e.g., to antimicrobial effects against *Streptococcus mutans* and inhibition of dental plaque formation in vitro [41, 42]. Additionally, lipophilic molecules like terpenes from essential oils damage the bacterial cell membrane [18]. The results of live/dead staining and metabolic activity are in line with these findings, as they clearly show an antibacterial mechanism. A killing of bacteria by REPHA-OS^®^ can indeed be assumed from the concentrations tested in this study onwards. As discussed above, the effect cannot be attributed to the included ethanol, but to the herbal ingredients. This correlates with results of the previous study conducted in our group, were a synergistic effect of medicinal plant extracts and essential oils was demonstrated [21]. The reason for the lower MIC in this study (5%) than in the previous one (10%; [21]) may be due to the different experimental setups. In an agar dilution assay, bacteria only lie on the active substance, in a broth solution the cells are completely surrounded by it. Besides the antimicrobial effect by the majority of the individual ingredients, most of the ingredients (e.g., mhyrre, bloodroot, clove, eucalyptus peppermint, anise) additionally dispose anti-inflammatory activities [43–48] and in some cases (peppermint, anise) analgesic effects [49, 50]. With this combination, the product may provide a holistic effect for the treatment of periodontal disease.

This study further showed that REPHA-OS^®^ not only has an antimicrobial effect on individual bacterial species, but also on multispecies bacteria in the physiological relevant biofilm morphology. If bacteria are directly incubated with the product, a biofilm formation is effectively inhibited. If the product is added to mature biofilms, bacteria are killed within several minutes. A major characteristic of biofilms is their resistance to antibacterial substances. Often, 10–1000-fold higher concentrations are necessary to obtain the same effect as in the respective planktonic cultures [9]. Interestingly, the MBEC on mature biofilms (24 h old) was only slightly higher (10%) compared to the MIC or MBIC (both 5%). The medicinal plant extracts and the essential oils, thus, have to be able to effectively penetrate the biofilm, which strengthens the effectiveness of REPHA-OS^®^ as oral care product. However, at least after two hours of incubation, no dispersion or loosening of the biofilm structure could be observed. Therefore, REPHA-OS^®*^ is not able to remove already formed biofilms. For clinical application, this verifies that the oral spray is not able to replace mechanical biofilm removal rather than being used for supportive oral care.

For all experiments, a well-established multispecies biofilm model was used [22, 23, 51, 52]. This model was chosen because the distribution of the integrated bacterial species, *S.* *oralis*, *A. naeslundii*, *V.* *dispar* and *P.* *gingivalis,* reflects the situation in early oral biofilms and it has proven to be highly reproducible. When mature biofilms (24 h old) were treated with 5% REPHA-OS^®^ for five minutes, the number of viable cells was clearly reduced (Fig. 4d), but the distribution of the species *S.* *oralis*, *A. naeslundii* and *P. gingivalis* (in decreasing order) was almost maintained; with exception of *V. dispar*—initially the third most represented—which could not be detected anymore. After two-hour incubation with 5% REPHA-OS^®^, viable cells could only be detected for *S. oralis* with additional reduced amounts. The reduction of *V.* *dispar*, below the detection limit after just five minutes suggests that this species could be more susceptible to the contained substances than the other three species. The observation that *S. oralis* was the only detectable species after two hours of incubation may be due to the fact that it has previously accounted for the majority of the biofilm (> 90%), but also that it may not be as susceptible to REPHA-OS^®^. However, the detailed effect of the various ingredients of REPHA-OS^®^ on different bacterial species should be analyzed in further studies. Additionally, the influence of the product on bacterial survival and species distribution in vivo should be addressed.

## Conclusion

The present study analyzed the effect of the natural oral care product REPHA-OS^®^, based on medicinal plant extracts and essential oils, on different stages of oral multispecies biofilm formation in vitro. By determining bacterial growth and viability, a clear, significant antibacterial effect could be detected, with a MIC and MBIC of 5%, and a MBEC of 10%. This effect could not be related to a simple toxicity of the solvent ethanol. When analyzing the species distribution inside the biofilm, REPHA-OS^®^ first affected *V. dispar*, whereas the dominant *S. oralis* survived the longest. The strong antibacterial effect of REPHA-OS^®^ on all stages of oral multispecies biofilm formation combined with the additional anti-inflammatory and analgesic effects of single ingredients strengthens its application as preventive oral care product.

## Supplementary Information

**Additional file 1: Table S1.** Reaction components for qRT-PCR. **Table S2.** Primer pairs used in qRT-PCR to classify the different bacterial species. **Table S3**. Thermal cycler conditions for qRT-PCR. **Table S4.** Genome sizes, consulted accession numbers and the calculated genome weight used for individual cell count determination.

## Data Availability

The datasets used and/or analyzed during this study are available from the corresponding author on reasonable request.

## Abbreviations

ATP: Adenosine triphosphate
BHI: Brain heart infusion
BHI/VitK: Control group cultivated in Brain Heart Infusion supplemented with 10 µg/ml vitamin K
BHI/VitK red: Control group cultivated in water-reduced Brain Heart Infusion supplemented with 10 µg/ml vitamin K to cope for the dilution effect
DNA: Deoxyribonucleic acid
MBEC: Minimum biofilm eradication concentration
MBIC: Minimum biofilm inhibitory concentration
MIC: Minimum inhibitory concentration
OD_600_: Optical density at 600 nm
PBS: Phosphate buffered saline
PCR: Polymerase chain reaction
PMA: Propidium monoazide
