# Supplementary material for: Plant-based oral care product exhibits antibacterial effects on different stages of oral multispecies biofilm development in vitro
Source: BMC Oral Health. 2021 Apr 1;21:170. doi: 10.1186/s12903-021-01504-4 (PMC8015205; doi:10.1186/s12903-021-01504-4)
Supplement: Supplementary file 1 — Additional file 1: Table S1. Reaction components for qRT-PCR. Table S2. Primer pairs used in qRT-PCR to classify the different bacterial species. Table S3. Thermal cycler conditions for qRT-PCR. Table S4. Genome sizes, consulted accession numbers and the calculated genome weight used for individual cell count determination. [file 12903_2021_1504_MOESM1_ESM.docx]

**Supplementary Information**

**Plant-based Oral Care Product Exhibits Antibacterial Effects on Different Stages of Oral Multispecies Biofilm Development *In Vitro***

Nadine Kommerein^#^*, Almut Johanna Weigel^#^, Meike Stiesch^¶^, Katharina Doll^¶^

Department of Prosthetic Dentistry and Biomedical Materials Science, Hannover Medical School, Carl-Neuberg-Str. 1, 30625 Hannover, Germany

^#^ equally contributed

^¶^ equally contributed

*Corresponding author

Nadine Kommerein, Department of Prosthetic Dentistry and Biomedical Materials Science, Lower Saxony Center for Biomedical Engineering, Implant Research and Development (NIFE), Hannover Medical School, Stadtfelddamm 34, 30625 Hannover, Germany

Tel.: +49 511 532 1416

E-mail address: Kommerein.Nadine@mh-hannover.de

**Table S1.**  **Reaction components for qRT-PCR.**

| **Component** | **Volume** | **Final Concentration** |
| --- | --- | --- |
| 2x Mastermix iQ^™^ SYBR^®^ Green Supermix  (Bio-Rad, Hercules, California, USA) | 12.5 µl | 1x |
| Forward Primer | 0.5 µl | 0.2 µM |
| Reverse Primer | 0.5 µl | 0.2 µM |
| Water, PCR grade  (Roche Life Science, Penzberg, Germany) | variable | - |
| Template DNA | variable (1-40 ng) | 40 pg – 1.6 ng |
| **Total Volume** | **25 µl** |  |

**Table S2. Primer pairs used in qRT-PCR to classify the different bacterial species.**

| **Species** | **Primer pairs** | **Annealing temp. [°C]** | **Gene** | **Expected size [bp]** | **Ref.** |
| --- | --- | --- | --- | --- | --- |
| *S. oralis* | F: 5’ - TCC CGG TCA GCA AAC TCC AGC C - 3’  R: 5’ - GCA ACC TTT GGA TTT GCA AC - 3’ | 58 | *gtfR* | 374 | [1,2] |
| *A. naeslundii* | F: 5’ - CAA CGT CGA GGA GAT CCA GG - 3’  R: 5’ - TAT TGA GGA CCA CCT TGG CG - 3’ | 58 | *gyrA* | 215 | [2] |
| *V. dispar* | F: 5’ - TGG AGC AAA CCC GAG AAA CA - 3’  R: 5’ - TTC ACC GCA GTA TGC TGA CC - 3’ | 58 | *16S rRNA* | 104 | [2] |
| *P. gingivalis* | F: 5’ - AGG CAG CTT GCC ATA CTG CG - 3’  R: 5’ - ACT GTT AGC AAC TAC CGA TGT - 3’ | 56 | *16S rRNA* | 405 | [2,3] |

**References**

1. Hoshino T, Kawaguchi M, Shimizu N, Hoshino N, Ooshima T, Fujiwara T. PCR detection and identification of oral streptococci in saliva samples using gtf genes. Diagn Microbiol Infect Dis. 48. United States2004. p. 195-9.

2. Kommerein N, Stumpp SN, Musken M, Ehlert N, Winkel A, Haussler S, et al. An oral multispecies biofilm model for high content screening applications. PLoS One. 2017;12(3):e0173973. Epub 2017/03/16. doi: 10.1371/journal.pone.0173973. PubMed PMID: 28296966.

3. Ashimoto A, Chen C, Bakker I, Slots J. Polymerase chain reaction detection of 8 putative periodontal pathogens in subgingival plaque of gingivitis and advanced periodontitis lesions. Oral Microbiol Immunol. 1996;11(4):266-73. Epub 1996/08/01. PubMed PMID: 9002880.

**Table S3.**  **Thermal cycler conditions for qRT-PCR.**

| **Step** | **Temperature [°C]** | **Time [sec]** | **Cycles** |
| --- | --- | --- | --- |
| Pre-denaturation | 95 | 180 | 1x |
| Denaturation | 95 | 10 |  |
| Annealing | individual (see table S2) | 20 | 40x |
| Extension | 72 | 20 |  |
| Melting curve analysis | 60 | 6 | 150x |

**Table S4.** **Genome sizes, consulted accession numbers and the calculated genome weight used for individual cell count determination.**

| **Species** | **Accession number** | **Genome size [bp]** | **Genome weight [ng]** |
| --- | --- | --- | --- |
| *S. oralis* | NC_015291.1 | 1.96E+06 | 2.15E-06 |
| *A. naeslundii* | ALJK00000000.1 | 3.04E+06 | 3.33E-06 |
| *V. dispar* | NZ_ACIK00000000.2 | 2.11E+06 | 2.32E-06 |
| *P. gingivalis* | NC_015571.1 | 2.34E+06 | 2.57E-06 |
